# Occipital cortex is modulated by transsaccadic changes in spatial frequency: an fMRI study

**DOI:** 10.1038/s41598-021-87506-2

**Published:** 2021-04-21

**Authors:** Bianca R. Baltaretu, Benjamin T. Dunkley, W. Dale Stevens, J. Douglas Crawford

**Affiliations:** 1 Centre for Vision Research and Vision: Science To Applications (VISTA) Program, York University, Toronto, ON M3J 1P3 Canada; 2 Department of Biology, York University, Toronto, ON M3J 1P3 Canada; 3 Department of Diagnostic Imaging, Hospital for Sick Children, Toronto, ON M5G 1X8 Canada; 4 Neurosciences and Mental Health, Hospital for Sick Children Research Institute, Toronto, ON M5G 0A4 Canada; 5 Department of Medical Imaging, University of Toronto, Toronto, ON M5T 1W7 Canada; 6 Department of Psychology and Neuroscience Graduate Diploma Program, York University, Toronto, ON M3J 1P3 Canada; 7 School of Kinesiology and Health Sciences, York University, Toronto, ON M3J 1P3 Canada

**Keywords:** Neuroscience, Oculomotor system, Sensory processing, Visual system

## Abstract

Previous neuroimaging studies have shown that inferior parietal and ventral occipital cortex are involved in the transsaccadic processing of visual object orientation. Here, we investigated whether the same areas are also involved in transsaccadic processing of a different feature, namely, spatial frequency. We employed a functional magnetic resonance imaging paradigm where participants briefly viewed a grating stimulus with a specific spatial frequency that later reappeared with the same or different frequency, after a saccade or continuous fixation. First, using a whole-brain Saccade > Fixation contrast, we localized two frontal (left precentral sulcus and right medial superior frontal gyrus), four parietal (bilateral superior parietal lobule and precuneus), and four occipital (bilateral cuneus and lingual gyri) regions. Whereas the frontoparietal sites showed task specificity, the occipital sites were also modulated in a saccade control task. Only occipital cortex showed transsaccadic feature modulations, with significant repetition *enhancement* in right cuneus. These observations (parietal task specificity, occipital enhancement, right lateralization) are consistent with previous transsaccadic studies. However, the specific regions differed (ventrolateral for orientation, dorsomedial for spatial frequency). Overall, this study supports a general role for occipital and parietal cortex in transsaccadic vision, with a specific role for cuneus in spatial frequency processing.

## Introduction

The visual system tracks both low-level (e.g., orientation, spatial frequency) and high-level (e.g., objects, faces) components of our visual surroundings through space and time^1^, despite the interruption of several saccades (rapid eye movements) per second^2,3^. To do this, visual features must be encoded, retained, updated, and integrated across saccades^4,5^, through a process called transsaccadic perception^2,6–8^. As argued elsewhere^9–11^, transsaccadic perception likely incorporates mechanisms for both visual working memory^12,13^ and spatial updating^14,15^. However, the specific *neural* mechanisms for human transsaccadic feature perception are not well understood.

When saccades occur, both object locations and their associated features shift relative to eye position. It is well established that human posterior parietal cortex (PPC; specifically, the mid-posterior parietal sulcus) is involved in transsaccadic spatial updating, i.e., the updating of object *location* relative to each new eye position^16–20^. Recently, we found that inferior PPC (specifically, right supramarginal gyrus; SMG) is also modulated by transsaccadic comparisons of object orientation^21^. Specifically, when a circular grating was presented, followed by a saccade and then presentation of a grating with a different orientation, SMG showed *repetition suppression* (compared to presentation of the same stimulus in the same orientation). Conversely, a ventrolateral occipital area (‘putative V4′) showed *repetition enhancement*. Both observations suggest underlying cortical activity modulations specific to transsaccadic interactions of object orientation.

Consistent with these findings, transcranial magnetic stimulation (TMS) of PPC (just posterior to SMG) disrupted transsaccadic memory of multiple object orientations^22,23^, and TMS over occipital cortex disrupted gaze-centered updating of object orientation^24^. SMG activity was also modulated during transsaccadic updating of object orientation for grasp, along with other parietal sensorimotor areas. Whereas SMG was always saccade-modulated, the sensorimotor grasp areas were only saccade-modulated when a grasp was planned^25^. Functional connectivity analysis suggested that these areas also communicated with the frontal/supplementary eye fields, possibly providing the motor signal that drives the updating^23,25^. These findings implicate both occipital cortex and PPC in the transsaccadic updating of *both* object location and orientation.

Importantly, it is not known if these neural mechanisms generalize to other stimulus features. One might expect PPC to be involved in other aspects of transsaccadic feature memory and integration, because of its general role in spatial updating^17,26,27^; however, the specific mechanisms might differ. SMG seems to play a specialized role for high-level object orientation in various spatial tasks^28,29^. Likewise, extrastriate cortical areas show specialization for processing different features^30,31^. Thus, while SMG and ‘putative V4’ might play a general role in transsaccadic updating of all visual features, it is equally possible that the brain engages different cortical networks for transsaccadic processing of different features, as it does during prolonged visual fixations^32,33^.

**Figure 1:**
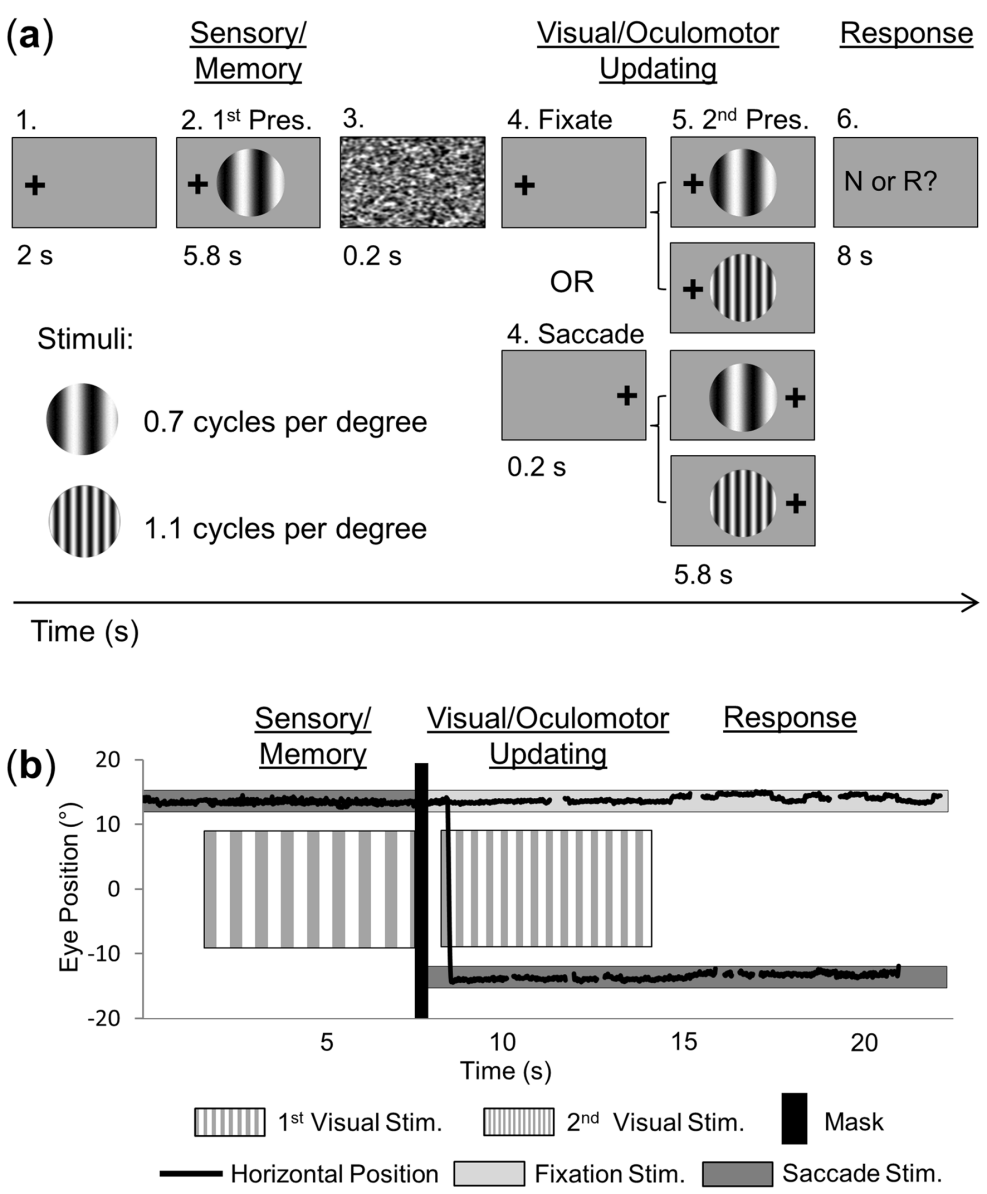
Experimental paradigm and eye movement traces. (**a**) An example trial is shown (0.7 cycles per degree, cpd, left fixation) with the four possible conditions: Fixation Same, Fixation Different, Saccade Same, and Saccade Different. Each 22 s trial had three major phases: (1) *Sensory/Memory* for the first presentation of the stimulus at one of the two possible spatial frequencies (0.7 or 1.1 cpd) while gaze could be to the left or right; (2) *Visual/Oculomotor Updating*, for the second presentation of the stimulus at the same spatial frequency (e.g., 0.7 cpd for first and second stimulus presentations; *Same* condition) or different (e.g., 0.7 cpd for first presentation and 1.1 cpd for second stimulus presentation, or vice versa; *Different* condition) while participants maintained fixation on the same cross (*Fixate* condition) or made a directed saccade (*Saccade* condition); and (3) *Response*, for the button press response period where an indication of whether the spatial frequency across the two stimulus presentations was the same (‘R’) or different (‘N’). **(b**) An example eye position trace (º) for an example fixation and saccade trial. In this figure, each of the two trials started with initial fixation on the right (13.5º from centre), then diverged after the initial presentation of the stimulus (1st visual stimulus) and mask (black vertical bar), whereby gaze remained fixed for the fixation trial or moved to the other fixation cross position (− 13.5º from centre) for the saccade trial, where it remained after the second stimulus presentation (2nd visual stimulus), and button press period.

**Figure 2:**
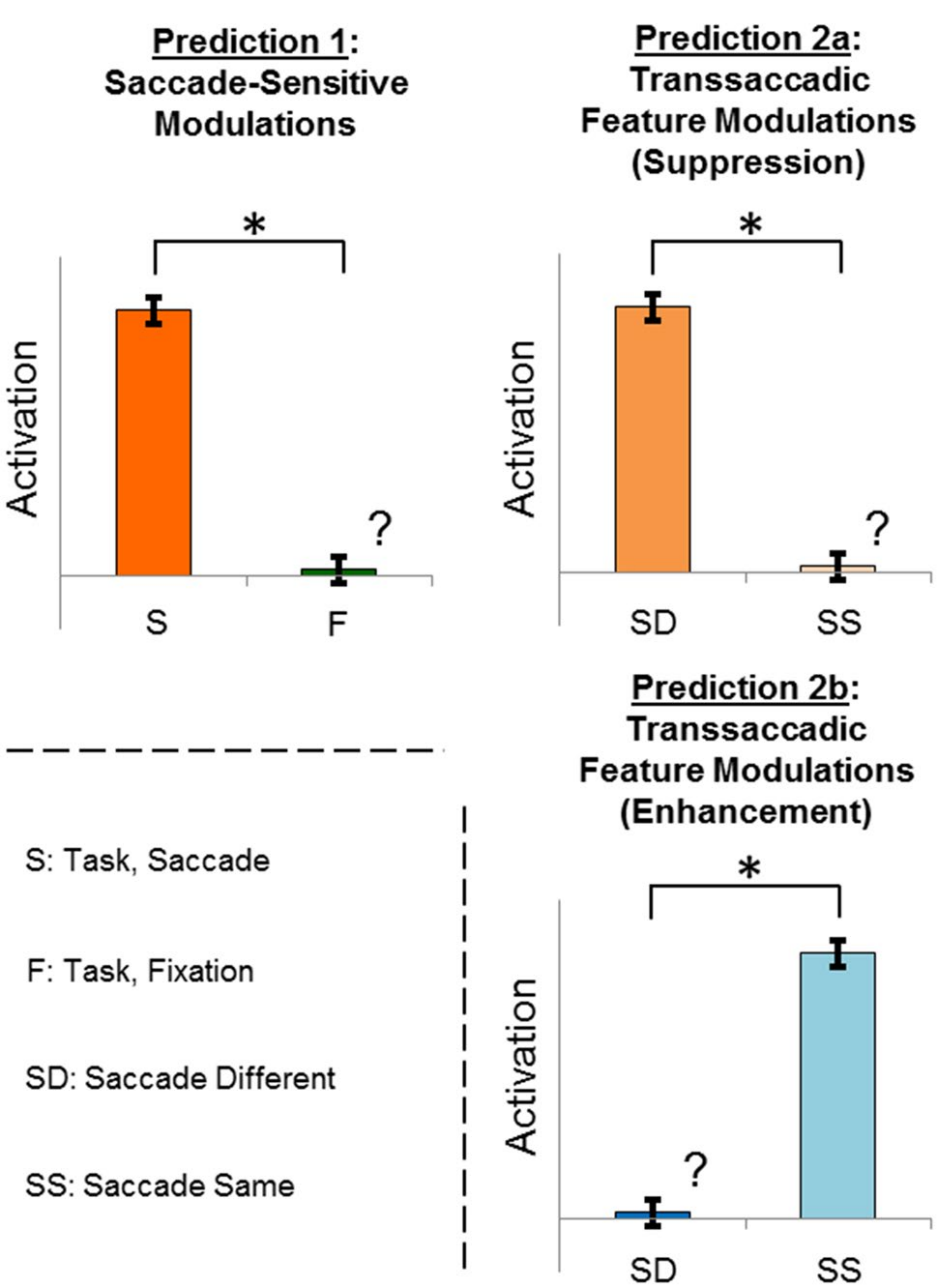
Predictions used to test saccade- and feature-specificity. Prediction 1: The saccade-related effects in this region should be larger in the *Saccade* condition than the *Fixation* condition, within the experimental task (i.e., (Saccade – Fixation); S, F, respectively). Prediction 2a: Parietal areas should show transsaccadic feature repetition *suppression*^21,25^*, i.e.,* a larger response for a change in spatial frequency than a repetition, following an intervening saccade (i.e., (Saccade/Different > Saccade/Same); SD, SS, respectively). Prediction 2b: Alternatively, occipital areas have been known to show transsaccadic repetition *enhancement*^21^, whereby there should be a larger response for a repetition in spatial frequency than for changes, following an intervening saccade (i.e., (Saccade/Same > Saccade/Different)) (Microsoft PowerPoint v16.44; www.microsoft.com).

To address this question, we used an event-related fMRI paradigm, similar to Dunkley et al.^21^, where participants briefly viewed two successive 2D spatial frequency grating stimuli, either while continually fixating the eyes, or interleaved by a saccade to the opposite side, and then judged whether the two gratings were the same or different. But here, we modulated spatial frequency, rather than orientation (Fig. 1a,b). Using a hypothesis-driven approach^25^, we first performed a whole-brain analysis to identify cortical regions that were modulated by saccades during the task (Fig. 2, prediction 1). As in our previous experiment^25^, we also compared these modulations to activation in a simple saccade task, to see if they were task-specific. We, then, tested if sites of peak activation within these regions showed feature-specific modulations (Fig. 2, prediction 2). Based on our previous experiments^21,25^, we expected repetition *suppression* in PPC (prediction 2a) and *enhancement* in occipital cortex (prediction 2b), whereas frontal cortex was not expected to show (directional) feature-related modulations. As a secondary issue, we also tested if these areas showed task specificity (or not) compared to our previous study^25^. Our results support the general role of occipital and parietal cortex in transsaccadic visual processing^21^, with similar parietal task specificity^25^, but suggest that different, more dorsomedial areas are involved for processing spatial frequency.

## Results

**Figure 3:**
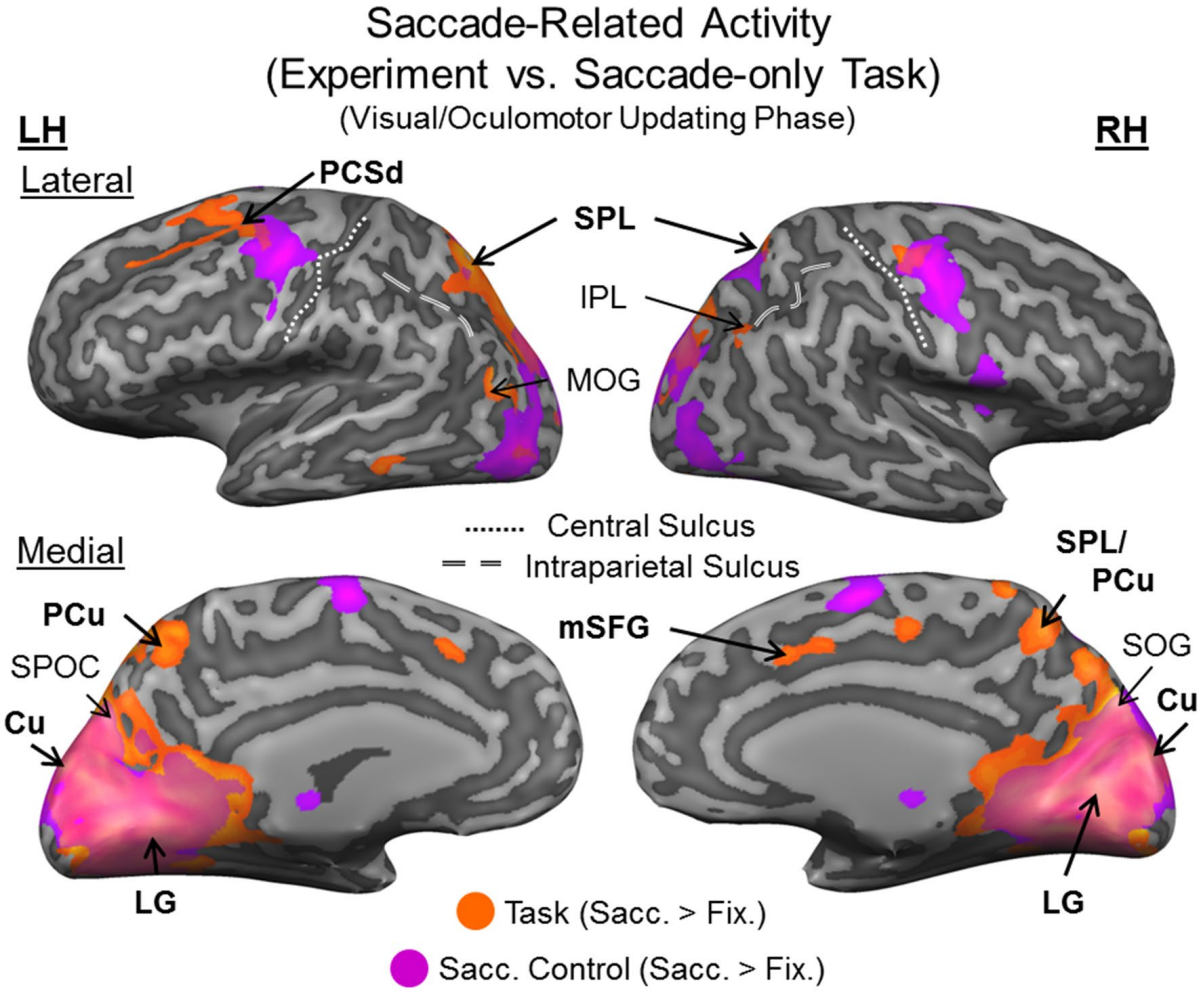
Saccade-related effects during experimental task versus independent saccade task. Voxelwise statistical maps from an RFX GLM for Saccade > Fixation in the experimental task (n = 15; orange; FDR (q < 0.05) and cluster correction) versus the control task (n = 12; fuchsia; FDR (q < 0.05) and cluster correction) are overlaid onto inflated cortical surface renderings of example participant (left hemisphere on the left, right hemisphere on the right; upper panels showing the lateral views, and lower panels showing medial views) (BrainVoyager QX v2.8; www.brainvoyager.com). There is substantial overlap in activity in medial occipital regions for task and saccade control (for example, cuneus, Cu, lingual gyrus, LG, and superior occipital gyrus, SOG). However, experimental task-specific saccade modulations can be observed in particular parietal (precuneus, PCu, and superior parietal lobule, SPL) and frontal saccade regions (medial superior frontal gyrus, mSFG—likely pre-supplementary eye field; dorsal precentral sulcus, PCSd—likely frontal eye field). (Bold regions are those that are subsequently tested against prediction 2).

**Figure 4:**
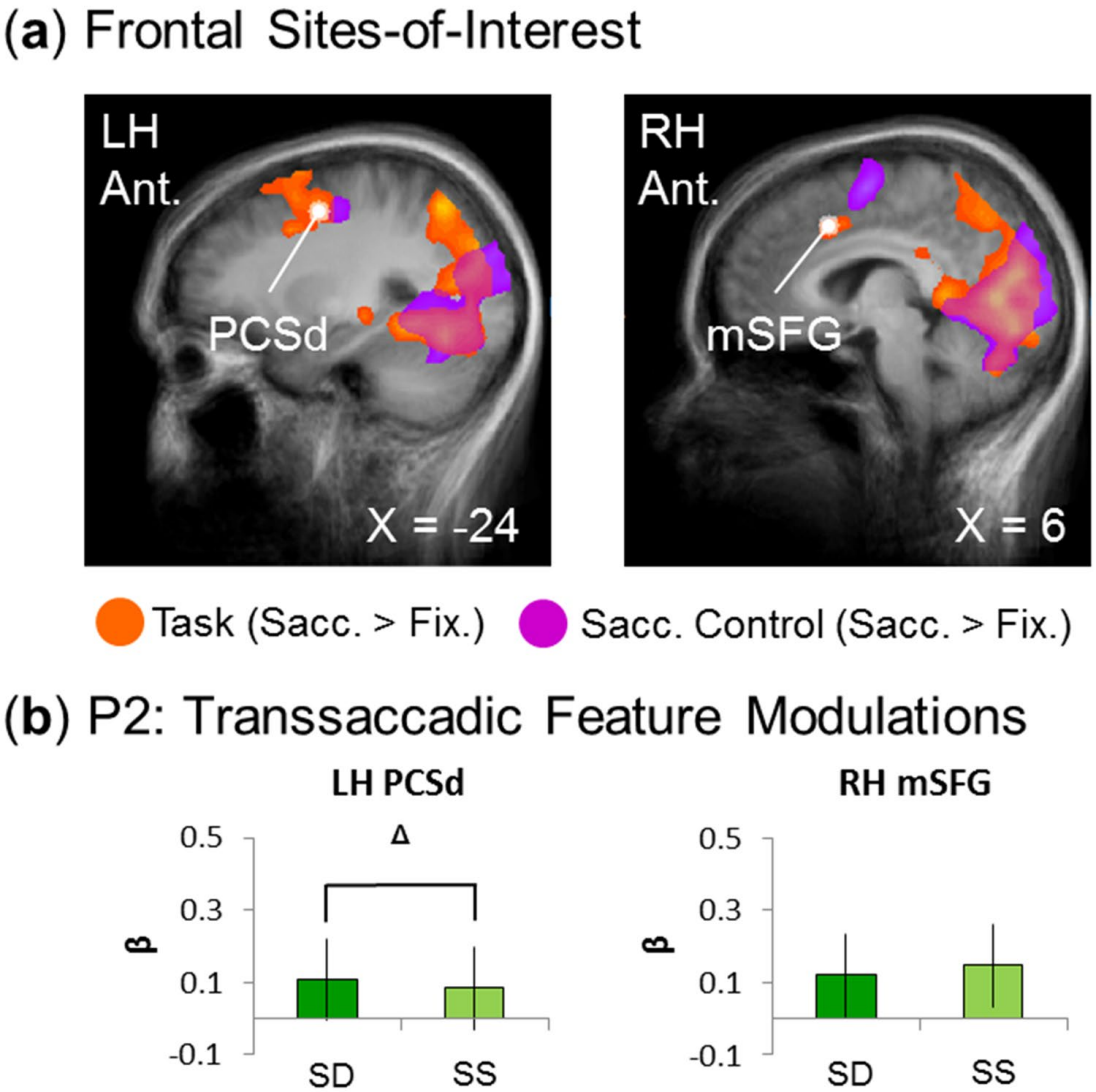
Testing feature sensitivity in frontal sites-of-interest. (**a**) The sites-of-interest in frontal cortex were localized and are visualized on the slices of the averaged brains of all (n = 15) participants. (These regions are superimposed onto activation maps from an RFX GLM for the experimental transsaccadic data (n = 15; orange) and from the separate saccade control task (n = 12; fuchsia) (BrainVoyager QX v2.8; www.brainvoyager.com). The white dots represent peak voxels of each frontal site-of-interest. The left panel shows the left dorsal precentral sulcus (PCSd) and the right panel shows the right medial superior frontal gyrus (mSFG). (**b**) We tested feature sensitivity in frontal regions, though with no particular directional hypothesis, for spatial frequency (experimental task data; n = 15). Results indicate that there is a trend toward statistical significance (*p* < 0.10) for feature sensitivity in left PCSd, with no effect in right mSFG. Overall, this supports a more general saccade-related role for frontal regions in transsaccadic tasks, like this one. Bar graphs show mean β-weights ± SEM analyzed using two-tailed repeated measures t-tests.

**Figure 5:**
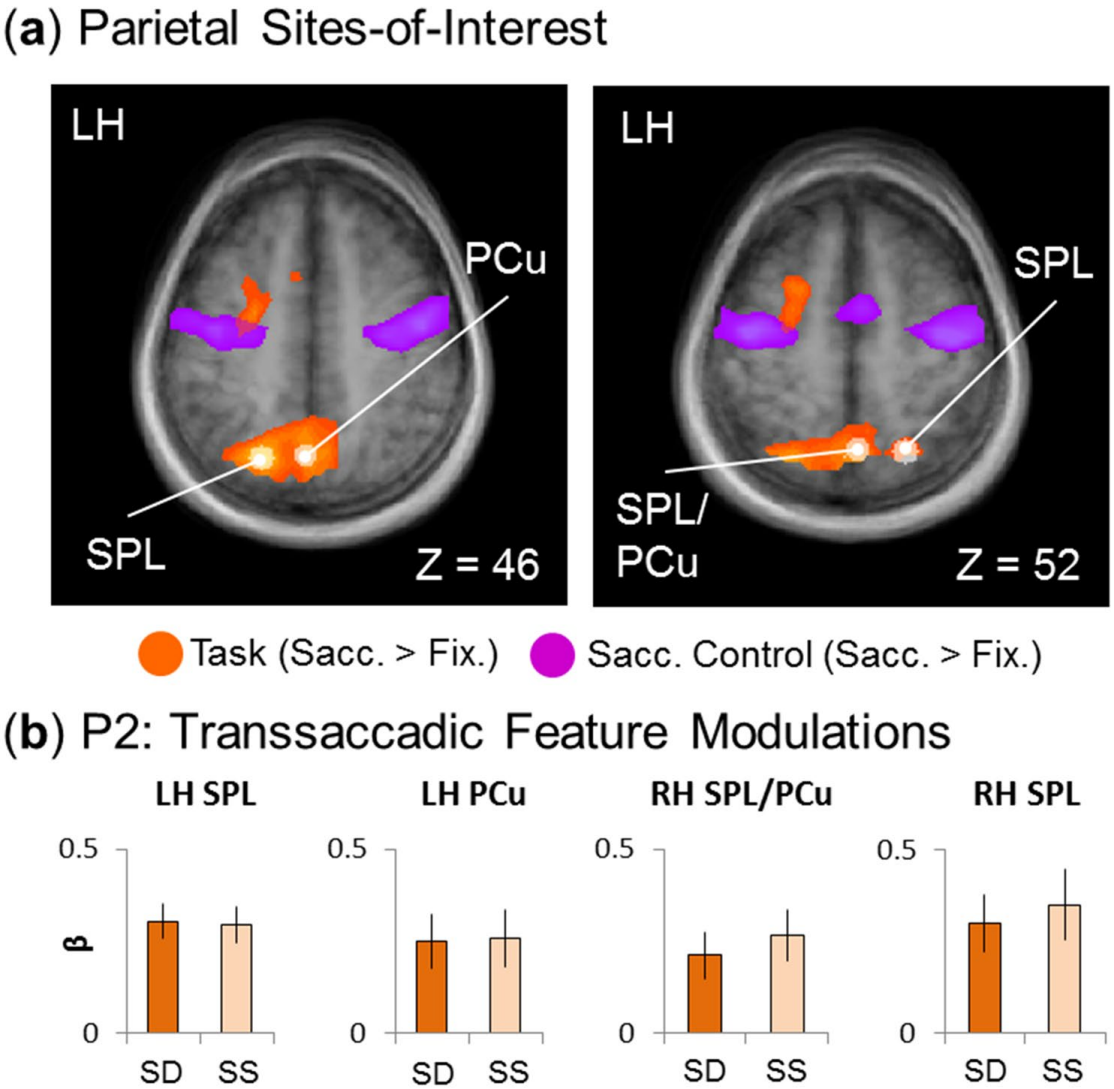
Testing feature specificity in parietal sites-of-interest. (**a**) The sites-of-interest within the parietal cortex were localized, the peak voxels of which are visualized in the transverse slices through the average brain of all (n = 15) participants via the white dots. (These regions are superimposed onto activation maps from an RFX GLM for the experimental transsaccadic data (n = 15; orange) and from the separate saccade control task (n = 12; fuchsia) (BrainVoyager QX v2.8; www.brainvoyager.com).) Spheres (radius = 5 mm) were created around the peak voxels and β-weights were then extracted and tested. The specific regions were centred on left superior parietal lobule (SPL), left precuneus (PCu), a region in right hemisphere spanning across SPL and PCu (SPL/PCu), and right SPL. (**b**) Feature specificity (Fig. 2, prediction 2a) was tested within the parietal sites-of-interest. For these sites to show this type of specificity, there is an expectation that there will be greater β-weights for the *Saccade/Different* than the *Saccade/Same* condition. Of the four regions, none shows this directional *suppression* effect. Bar graphs show mean β-weights ± SEM analyzed using one-tailed repeated measures t-tests.

**Figure 6:**
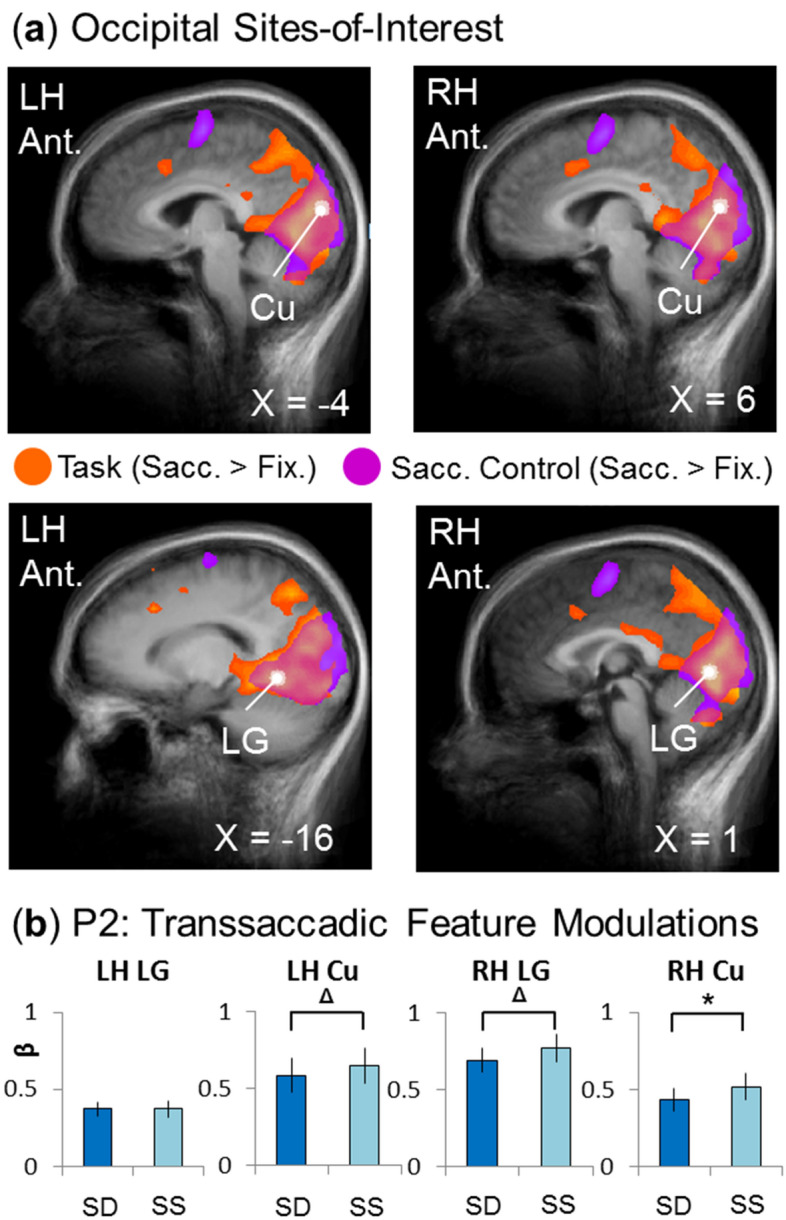
Testing feature specificity in occipital sites-of-interest. (**a**) The sites-of-interest within the occipital cortex were determined and are visualized on the slices of the averaged brains of all (n = 15) participants. (These regions are superimposed onto activation maps from an RFX GLM for the experimental transsaccadic data (n = 15; orange) and from the separate saccade control task (n = 12; fuchsia) (BrainVoyager QX v2.8; www.brainvoyager.com). The white dots represent the peak voxels of each site-of-interest. The top two panels show the location of left lingual gyrus (LG) and cuneus (Cu) (left and right, respectively), whereas the bottom two panels show the locations of the right LG and Cu (left and right panels, respectively). (**b**) Based on previous findings for enhancement within occipital regions (see main text), we tested prediction 2c (see Fig. 2) to determine if these occipital sites-of-interest show a feature-specific effect for spatial frequency. These effects would be consistent with greater β-weights for the *Saccade/Same* condition than for the *Saccade/Different* condition. Bar graphs show the extracted β-weights from each of the four regions, of which right Cu shows a statistically significant effect (*p* < 0.05) and left Cu and left LG show a trend toward significance (*p* < 0.10). Bar graphs show mean β-weights ± SEM analyzed using one-tailed repeated measures t-tests.

Overall, our task (Fig. 1) produced widespread activation in brain areas related to vision, visual memory, and eye movements during the initial *Sensory/Memory Phase* (Fig. S1) and *Visual/Oculomotor Updating Phase* (Fig. S2). To test our hypotheses, we focused on the *Visual/Oculomotor Updating* phase of our task, i.e. the period after the first stimulus presentation, starting at the time when a saccade occurred and a second stimulus (either Same or Different) appeared (Fig. 1a). As in our recent study^25^, we used an analysis pipeline that began with a whole-brain voxelwise contrast to identify regions of interest, followed by further hypotheses testing on peak sites-of-interest. In this dataset, saccade modulations were more robust than feature modulations, so we used a *Saccade* vs. *Fixation* contrast (Fig. 3) to identify regions that qualify for prediction 1 (Fig. 2). This was then used to localize specific sites-of-interest to test prediction 2 (Fig. 2), i.e., feature interactions (Figs. 4, 5, 6). Additionally, we used a simple saccade motor task to test if these areas are automatically driven by saccade signals (as observed previously in SMG), or are only activated by saccades in a task-specific fashion^25^. An a priori power analysis suggested that 14 participants were required for the voxelwise contrasts used in this pipeline (see Methods: Power analysis). To obtain this level, we continued testing participants (21 in total), until 15 of these passed our behavioural inclusion criteria for fMRI analysis (see Methods: Behavioural data and exclusion criteria).

### Prediction 1: saccade modulations

#### Whole-brain analysis

The first step in our analysis was a whole-brain voxelwise contrast (*Saccade* > *Fixation*; Figs. 2, 3) on fMRI data derived from the *Visual/Oculomotor Updating* phase of our experimental task. The resulting group data are shown in Fig. 3 (orange; n = 15), overlaid on a representative anatomical scan in ‘inflated brain’ coordinates (note that while visually convenient, this convention results in small spatial distortions). This contrast revealed extensive cortical activation spanning occipital and parietal cortex, with some additional activation in frontal cortex. Also shown is the *Saccade* > *Fixation* contrast (fuchsia) from an independent saccade control task (n = 12), where participants simply made saccades back and forth between two points (see Methods). Note that, overall, saccade modulations were much more widespread in the experimental task, suggesting that additional saccade interactions were required for the more complex visual processing and response required in this task^25^.

#### Sites-of-interest

The second step in this analysis was to determine specific cortical coordinates for our hypothesis tests. To do this, we localized the sites of peak activation within the occipital and parietal lobes from our experimental transsaccadic task. This resulted in (1) two frontal sites: right medial superior frontal gyrus, mSFG, and left dorsal precentral sulcus, PCSd, (2) four parietal sites: bilateral superior parietal cortex (SPL) and bilateral precuneus (PCu), and (3) four occipital sites: bilateral lingual gyrus (LG) and cuneus (Cu). These sites are shown in Figs. 4a, 5a, and 6a, respectively, overlaid on anatomical slices (less susceptible to distortions). Coordinates and supporting references for these sites are shown in Table 1. Note that, by definition, each of these sites passed our prediction 1 (Saccade > Fixation), so were next tested on prediction 2.

**Table 1:** Regions, Talairach coordinates, and statistical results for tests of Prediction 2 (t-value, *p *value, and effect size) for parietal and occipital sites-of-interest from the *Visual/Oculomotor Updating* phase.

Site-of-interest	Talairach coordinates			Testing prediction 2			References
	x	y	z	T-value	*P* value	Effect Size	
**Frontal areas**							
LH dorsal precentral sulcus	− 24	− 3	46	2.145	0.097	0.46	^23^
RH medial superior frontal gyrus	6	14	40	2.145	0.363	0.24	^71^
**Parietal areas**							
LH superior parietal lobule	− 21	− 64	46	0.384	0.35	0.099	^25^
LH precuneus	− 3	− 63	47	− 0.190	0.57	0.049	^53^
RH superior parietal lobule/precuneus	2	− 60	54	− 1.098	0.86	0.28	^53^
RH superior parietal lobule	21	− 61	52	− 1.599	0.93	0.41	^25^
**Occipital areas**							
LH lingual gyrus	− 16	− 55	− 6	0.0731	0.53	0.019	^72^
LH cuneus	− 4	− 85	14	− 1.443	0.085	0.37	^73^
RH lingual gyrus	1	− 71	1	− 1.663	0.059	0.43	^72^
RH cuneus	6	− 79	15	− 1.960	0.035	0.51	^73^

### Prediction 2: transsaccadic feature modulations

#### Frontal cortex

Figure 4a provides an overview of the frontal cortex data used to test our hypotheses, showing *Saccade* > *Fixation* modulations during the updating portion of our experimental transsaccadic task (orange) and for reference, the same contrast from the saccade control task (fuchsia). The white arrows/dots indicate the sites of peak activity used for further testing including right mSFG and left PCSd, likely corresponding to pre-supplementary eye fields and frontal eye fields respectively (Table 1). Caudal PCSd appears to overlap with a region of control task activation, whereas mSFG is anterior to another region of the control task (Figs. 3, 4a). For further hypothesis testing, we used BrainVoyager (BrainVoyager QX v2.8, Brain Innovation) to create spheres (radius = 5 mm) around these peaks and then, extracted β-weights from those spheres to test our feature modulation prediction^﻿2﻿3,34,35^. Since transsaccadic feature modulations were not expected in frontal cortex, we applied a two-tailed repeated-measures t-test (Prediction 2a/2b, n = 15). There was a trend toward feature modulation in left PCSd (Fig. 4b; p < 0.10; Table 1), but neither site reached significance (Fig. 4b; Table 1), consistent with the notion that these frontal areas are primarily involved in the saccade motor aspects of such tasks^﻿2﻿3,25,36^.

#### Parietal cortex

Figure 5 follows the same conventions as Fig. 4, but shows our parietal data. Again, Fig. 5a shows the *Saccade > Fixation* contrast and peak sites from the experimental transsaccadic task (orange), as well as the saccade control contrast (fuchsia). The white arrows/dots indicate the sites of peak activity used for further testing of bilateral SPL and bilateral PCu, with right PCu bordering on SPL (Table 1). None of these sites appears to overlap with activation in the control task (Fig. 3, 5a), suggesting task-specific saccade modulations. Figure 5b shows the mean β-weights used for testing prediction 2 (Fig. 2; n = 15). Contrary to this prediction, none of these areas showed feature repetition *suppression* (Table 1). Overall, our parietal sites were modulated by saccades in a task-specific manner, and did not show transsaccadic feature modulations.

#### Occipital cortex

Figure 6 follows the same conventions and methods as Figs. 4 and 5, except showing our occipital cortex data. Again, Fig. 6a shows the *Saccade* > *Fixation* contrast and peak sites from the experimental transsaccadic task (orange), as well as the saccade control contrast (fuchsia). The white arrows/dots indicate the sites of peak activity used for further testing (bilateral LG and Cu; Table 1; extracted from experimental task data). In this case, all of these sites overlapped with the activation produced by the saccade control task (Figs. 3, 6a). Figure 6b shows the mean β-weights used to test prediction 2 (Fig. 2; *Transsaccadic Frequency Modulations (Enhancement)*). Here, we compared *Different* (SD) versus *Same* (SS) spatial frequency in the experimental *Saccade* task (n = 15). Right Cu showed significantly greater modulation in the *Same* condition (Table 1), with the other regions showing a similar trend (right LG and left Cu) or no feature-specific effect (left LG). In summary, our occipital sites showed general saccade modulations, and most showed repetition *enhancement*, i.e., they showed the properties expected for transsaccadic feature interactions^21,25^.

## Discussion

The goal of this study was to determine whether the involvement of the cerebral cortex in transsaccadic updating of visual location and orientation generalizes to other object features, such as spatial frequency. Overall, frontoparietal cortex showed saccade modulations that appeared to be largely task-specific, but did not show significant transsaccadic modulation for spatial frequency. In contrast, occipital cortex showed general saccade modulations, and transsaccadic feature modulations (repetition *enhancement*). Of the individual sites tested, only right cuneus passed (with statistical significance) both of our predefined criteria for putative transsaccadic updating^25^.

### Role of frontoparietal and occipital cortex in transsaccadic updating

Previous studies have implicated frontal^23^, parietal^19,20,25^, and occipital^20,24^ cortex in various aspects of transsaccadic processing for visual location and orientation. The current results extend these findings to spatial frequency processing, and show both similarities and differences. It has been proposed previously that frontal cortex (i.e., frontal eye fields and perhaps supplementary eye fields) are involved in producing a saccade efference copy that projects to the visual system for functions like transsaccadic updating^23,36^. We did not find anything inconsistent with that idea. First, the areas we found were at (or near) those classical oculomotor structures, with mSFG just anterior to the coordinates for supplementary eye fields and PCSd overlapping with the coordinates for the frontal eye fields (Table 1). Second, the latter region overlapped partially with activation in our saccade control task (Figs. 3, 4a). Finally, this region did not show significant transsaccadic feature modulations for either orientation^25^ or spatial frequency, so it may not be directly involved in those processes.

It has previously been shown that PPC areas, specifically intraparietal sulcus and adjacent portions of inferior PPC, are involved in transsaccadic processing of object location and orientation, respectively^17–20,22,25^. In some respect, the current results are similar to our previous grasp orientation results^25^: the PPC saccade modulations identified here did not overlap with classic saccade motor areas^36–38^ or the activation observed in our saccade control task (Fig. 3, 5a). In other words, they appeared to be largely task-specific, as observed in grasp areas in our previous study^25^. However, instead of the saccade modulations observed in SMG in our previous orientation studies, we found modulations in more dorsomedial superior parietal and precuneus locations. Further, we did not observe significant feature modulations in any of these sites. This suggests that SMG may be specialized for transsaccadic orientation processing, and the other PPC areas are related to other aspects of the task (discussed in more detail below).

Other studies have implicated occipital cortex in gaze-centered remapping^16,18,31^ and transsaccadic orientation processing^20,24^. Here, all of the occipital sites that we investigated showed saccade modulations in both our experimental and control tasks. Second, occipital cortex showed transsaccadic feature repetition *enhancement*, which reached significance in right cuneus (with trends in left cuneus and right lingual gyrus; Fig. 6b). Thus, of all the sites we examined, only cuneus met our full criteria (general saccade modulation and feature modulation) for transsaccadic feature processing. In this respect, cuneus shows similar properties to SMG^21,25^, except that it seems to be involved in spatial frequency processing rather than orientation processing.

Finally, the areas that showed transsaccadic feature modulations were predominantly located in the right hemisphere (Fig. 6b), consistent with the lateralization observed previously for spatial attention^39,40^, spatial updating^41^, and transsaccadic orientation processing^21,25^. But, again, the specific cortical regions involved were different: in contrast to transsaccadic orientation processing areas (SMG, ‘V4’), the activity described here was located in more dorsomedial sites such as cuneus.

### Relation to other parietal and occipital functions

Clearly, the cortical regions identified here are not only involved in transsaccadic visual processing, but their other roles seem complementary. Ventral cuneus and lingual gyrus collectively contain extrastriate cortex^42^, which receives visual input from further upstream, subcortical thalamus and is involved in virtually visual functions, including spatial frequency processing^43^. Our specific cuneus site appears to be dorsal to V1, likely in human V3 (Table 1). V3 receives input from V1, projects to both the dorsal and ventral stream areas^44^ and is responsive to processing global features such as motion and patterns^45–47^. Given its involvement in transsaccadic location remapping^16,31^, V3 seems to be an ideal candidate for transsaccadic processing of complex visual features.

In general, precuneus is associated with complex visuospatial transformations^48–50^. Dorsal precuneus is associated with reaching^51–53^, saccades^54,55^, and visual memory^56^, whereas SPL is associated with grasp formation. These areas may have been activated for the manual response^57^. Whereas SPL showed transsaccadic feature modulations in our previous grasp orientation study^25^, they did not show feature modulations in the current study. This makes sense in light of the details of the task. First, spatial frequency is less relevant for grasp formation than object orientation. Second, in the current study, feature-related grasp shaping was not required, only a button press.

### Why is transsaccadic perception feature- and task-dependent?

As noted above, our previous experiments showed transsaccadic modulations for object orientation in inferior parietal cortex (specifically SMG) along with some task-dependent extrastriate^21^ and superior parietal areas^25^. Why would the sites and networks observed for transsaccadic spatial frequency processing in the current study be so different? In part, it is likely that transsaccadic perception builds on computations already used during fixation, which are themselves feature- and task-dependent^21,58–60^, especially in the higher-level visual areas activated in our tasks^61,62^. At early levels like V1, there is clear multiplexing of orientation and spatial frequency^63,64^, but at higher levels these features may tap into entirely different processes. As noted above, a change in orientation is relevant for spatial processing and actions like grasping^25,65^, whereas a change in spatial frequency can denote, for example, changes in higher level cognitive processes such as perceived identity and affordance, evoking very different cortical mechanisms^66^. Conversely, it is likely that more automatic bottom-up aspects of transsaccadic integration (such as the automatic integration of motion signals across saccades)^67^ involve different mechanisms again, perhaps primary visual cortex^16,31^. Thus, the notion of a dedicated ‘transsaccadic perception centre’ is likely naïve: transsaccadic vision, not prolonged fixation, is normal vision, and has likely developed different and nuanced mechanisms, depending on feature and task details.

## Conclusion

In this fMRI study, we set out to explore the cortical mechanism for transsaccadic processing of spatial frequency, with emphasis on the role of PPC and occipital cortex. It has been shown previously that PPC and occipital cortex show both saccade and feature modulations during transsaccadic processing of object orientation. The modulations found here showed several similar properties, in terms of saccade specificity, laterality, and occipital repetition enhancement. However, whereas SMG and putative V4 were implicated in orientation processing^21,25^, we found activation for different (more dorsomedial) areas for spatial frequency processing, perhaps with a special role for right cuneus. It remains to be seen how these functions extend to other object properties, such as shape, and how they are combined for more real-world tasks. Overall, these findings support the role of parietal and occipital cortex in transsaccadic vision, but suggest that different cortical networks are recruited for transsaccadic processing of different features.

## Materials and methods

### Participants

We tested 21 (human) participants from York University (Toronto, Canada) of whom 15 met our inclusion criteria for analyses (see Behavioural analysis and exclusion criteria), thus exceeding the requirement for sufficient statistical power (see Power analysis)^68^. These 15 individuals (average age: 26.6 ± 4.3 years; age range: 21–37; 11 females and 4 males; all right-handed) had no neurological disorders and normal or corrected-to-normal vision. Informed consent was obtained from each participant; all participants were remunerated for their time. We confirm that the experimental protocol involving human participants was approved by and in accordance with guidelines of the York University Human Participants Review Subcommittee.

### Experimental set-up

Participants passed initial MRI safety screening and were then instructed on the experimental task. Participants practiced before taking part in the experiment. Once they felt comfortable with the task, they assumed a supine position on the MRI table, with their head resting flat within a 32-channel head coil. An apparatus, holding a mirror to reflect images from the screen in the MRI bore, was attached to the head coil. An MRI-compatible eye-tracker (iViewX, SensoMotoric Instruments) was also attached to the apparatus in order to record the position of the right eye. Participants held an MRI-compatible button box in their right hand. Their right index and middle fingers rested on two buttons, used to provide the task responses when presented with the ‘go’ cue (see General paradigm).

### Stimuli

During the experiment, participants were presented with an 18° stimulus that contained a vertical sine-wave grating pattern, averaged to the mean luminance of the screen. Stimuli were presented in the centre of the screen on a light gray background (MATLAB; Mathworks, Inc.). The two spatial frequencies that were tested were 0.7 or 1.1 cycles/degree (cpd) (see Fig. 1a).

### General paradigm

In order to identify the cortical activity correlates of transsaccadic perception of spatial frequency, we used a modified 2 (Gaze: Fixate or Saccade) × 2 (Spatial Frequency: 0.7 or 1.1 cpd) slow event-related fMRI design^21^. Here, we modulated the spatial frequency (repeated or changed within the same trial; *‘Same’* and *‘Different’* conditions, respectively) and/or position of the eyes (continued fixation of gaze or a saccade was produced; *‘Fixation’* and *‘Saccade’* conditions). This resulted in four main conditions: (1) Fixation/Same, (2) Fixation/Different, (3) Saccade/Same, and (4) Saccade/Different. These were randomly intermingled and repeated four times within each run; there were six runs in total for each participant.

### Trial sequence

Each trial was composed of three main phases: (1) an initial presentation of the stimulus, which requires sensory processing and working memory storage (‘*Sensory/Memory’* phase); (2) a second presentation of the stimulus, which requires sensory processing and (oculomotor) updating (for saccades) (‘*Visual/Oculomotor Updating’* phase); and (3) a response period, where a button press was made to indicate if the spatial frequency of the stimulus presentations was the same or different (*‘Response’* phase). The sequence of events in each trial (Fig. 1a) began with a 2 s fixation period, with the fixation cross presented at one of two possible positions (13.5° to the left or right of centre, or 4.5° to the left or right of the central stimulus). The stimulus was then presented in the centre for 5.8 s, followed by a 200 ms static noise mask. Once the mask disappeared, a fixation cross appeared for 200 ms at the same position (*Fixation* condition) or the other fixation position (*Saccade* condition) (see Fig. 1b for eye position trace). The central stimulus was presented a second time for 5.8 s at the same spatial frequency (*Same* condition) as in the *Sensory/Memory* phase or at the other spatial frequency (*Different* condition). Finally, after the fixation cross and stimulus disappeared, a written prompt (‘R or N?’) appeared for 8 s in the same location as the fixation cross, prompting participants to indicate via button press whether the spatial frequency of the two stimulus presentations was the same (R) or different (N).

Each trial sequence lasted 22 s in total. The four main conditions were repeated four times per run, resulting in 16 trials per run. Each run started and ended with a 16 s period of central fixation, serving as a baseline. Overall, each run lasted 6 min 24 s.

### Saccade control task

We ran a separate saccade control task in order to identify regions of the brain that respond to saccade production (over those that respond to fixations only). The sequence of events began with an 8 s fixation period of a central white cross on a black background. This was followed by a period of directed saccadic eye movements between two fixation crosses, randomly from left to right across trials, for 8 s. The pattern of fixation followed by saccades was repeated 16 times per control run, lasting ~ 4 min 16 s for each of two runs.

### MRI parameters

A 3 T Siemens Magnetom TIM Trio MRI scanner at the York MRI Facility was used to acquire fMRI data. An echo-planar imaging (EPI) sequence (repetition time [TR] = 2000 ms; echo time [TE] = 30 ms; flip angle [FA] = 90°; field of view [FOV] = 240 × 240 mm, matrix size = 80 × 80, in-plane resolution = 3 mm × 3 mm; slice thickness = 3 mm) was acquired in ascending, interleaved order for each of the six functional runs and for the two separate saccade localizer runs. Thirty-three contiguous slices were acquired per volume for a total of 192 volumes of functional data in each experimental run, and 128 volumes of data for each saccade control run. A T1-weighted anatomical reference volume was obtained for each participant using an MPRAGE sequence (TR = 1900 ms; FA = 256 mm × 256 mm; voxel size = 1 × 1 × 1 mm^3^). 192 slices were acquired per volume of anatomical data.

## Analysis

### Power analysis

To determine the appropriate number of participants to provide a sufficient effect size and level of power, we used results from the most recent, relevant findings^25^. Specifically, we used the effect size (0.887, Cohen’s d) from the most relevant region of activation in parietal cortex (i.e., SMG) and applied the following properties: (1) two-tailed t-test option, (2) an α value of 0.05, and (3) a power value of 0.85. Using G*Power 3.1^69^, we determined that a minimum of 14 participants would be required to achieve an actual power value of 0.866. As noted above, we tested a total of 21 participants in order to exceed this minimal number, after application of exclusion criteria in the next section.

### Behavioural data and exclusion criteria

In our previous studies^21,25^, we found that experiments such as this are highly sensitive to head motion. Due to excessive head motion, data were excluded from analyses on the basis of two criteria: (1) presence of head motion over 1 mm/degree and/or (2) any abrupt motion of the head over 0.5 mm/degrees. If more than 50% of all data (i.e., at least 3 runs out of the total 6 runs for a given participant) were removed from analysis, the entire data set from that participant was removed. On this basis, data from six participants were removed. From the remaining 15 participants, one run was removed from data analysis for each of two participants, and two runs were removed for each of another two participants, for a total of six runs (6.7%).

Eye-tracking and button-press data were analyzed post-image acquisition in order to determine whether the task was completed correctly or not. Eye position data (e.g., Fig. 1b) were inspected visually to confirm that the eye fixated on the fixation crosses (within a region of 2º of fixation) and/or moved to the correct saccade location when prompted to do so in all trials. Button press responses were also inspected offline to ensure that participants responded correctly to the *Same*/*Different* condition trials. On these bases, 41 trials were excluded from further analysis (across all participants, a maximum of four trials were removed for any given participant, with a mode of one; 3.1% of the remaining data). Overall, accuracy of the 15 participants included in the final data analysis was 97.4% ± 3.1%. Separated by condition type, accuracy was (1) 95.3% ± 5.4% for the Fixation/Same condition, (2) 98.1% ± 3.1% for the Fixation/Different condition, (3) 96.9% ± 3.7% for the Saccade/Same condition, and (4) 97.8% ± 2.7% for the Saccade/Different condition. A repeated-measures ANOVA showed no main effect of eye movement (F_1,14_ = 0.485, *p* = 0.489), no main effect of feature (F_1,14_ = 3.278, *p* = 0.076), nor interaction (F_1,14_ = 0.950, *p* = 0.334). Only data for correct trials were included in all further analyses.

### Functional imaging data: experimental

To model the fMRI BOLD response, we used a general linear model (GLM) analysis. In this model, a standard two-gamma haemodynamic response function (BrainVoyager QX 2.8, Brain Innovation) was convolved with predictor variables^21^. We had five major classes of predictors for each trial: (1) a baseline predictor (“Baseline”), corresponding to the first and last 16 s of each run; (2) “Fixate”, which represented initial trial fixation (either left or right); (3) “Adapt”, which modeled the activity in response to the first stimulus presentation; (4) “Fixate/Same”, “Fixate/Different”, “Saccade/Same”, or “Saccade/Different” to model activity in response to the second stimulus presentation in one of the four main conditions; and 5) “Response”, which modeled the activity for the button press response period. GLMs were generated using the eight predictors per run for each participant (BrainVoyager QX 2.8, Brain Innovation).

Preprocessing of functional data from each run for all participants included slice scan-time correction (cubic spline), temporal filtering (for removal of frequencies < 2 cycles/run), and 3D motion correction (trilinear/sinc). Anatomical data were transformed to Talairach space^70^. Functional data were coregistered using gradient-based affine alignment (translation, rotation, scale affine transformation). Lastly, the functional data were spatially smoothed using a Gaussian kernel with a full width at half maximum of 8 mm.

Using the random-effects (RFX) GLM with all of the runs of all of the remaining 15 participants, we performed two major types of analyses: (1) site-of-interest prediction testing and (2) voxelwise contrasts (see *Hypothesis testing: Analysis and statistical considerations* and *Voxelwise map contrasts: Analysis* *and statistical considerations* for details).

### Functional imaging data: saccade control task

Each run of the saccade task had 16 repetitions of the fixation trials (8 s of central fixation) and saccade trials (8 s of directed saccades). Each trial type was coded by an 8 s predictor (“Fixation” for the fixation trials, and “Saccade” for the saccade trials). These predictors were convolved with the standard two-gamma haemodynamic response function (BrainVoyager QX 2.8, Brain Innovation). Preprocessing of functional and anatomical data occurred as for experimental data (see previous).

On the basis of behavioural data analysis (i.e., excessive motion > 1 mm), data from three participants were removed, leaving saccade control task data for 12 participants. These data were used in the prediction testing in order to identify regions related to the production of saccades.

### Voxelwise map contrasts: analysis and statistical considerations

We conducted voxelwise contrasts on data from the 15 participants included in the analysis for experimental task, and separately on the remaining 12 participants for the saccade control task. For analysis purposes, we divided the experimental task data into the *Sensory/Memory* phase (when participants saw and initially remembered the stimulus) and *Visual/Oculomotor Updating* phase (when saccades occurred and the stimulus reappeared). For all (voxelwise) contrasts, we first applied a False Discovery Rate (FDR) of q < 0.05, followed by cluster threshold correction (BrainVoyager QX v2.8, Brain Innovation) to our contrasts. This included the *Saccade* > *Fixation* contrast shown in Figs. 3, 4a, 5a, and 6a (orange), and the contrasts shown in Supplementary figures (*Sensory/Memory* phase activity > Baseline, Fig. S1; *Visual/Oculomotor Updating > Sensory/Memory* contrast, Fig. S2).

### Hypothesis testing: analysis and statistical considerations

In order to localize our site-of-interest testing, we used a similar approach to site localization as in Song and Jiang^34^; Baltaretu et al.^25^; and Tsushima et al.^35^ by first applying a whole-brain contrast of interest to only our experimental task data (n = 15): Saccade – Fixation (FDR with q < 0.05, followed by cluster threshold correction) (Fig. 3a). We then localized the peaks of our a priori predicted frontal, parietal and occipital activations and then used BrainVoyager (BrainVoyager QX v2.8, Brain Innovation) to create 5 mm-radius spheres surrounding the peak voxels (Table 1). Finally, we extracted the mean β-weights across voxels within each sphere for the experimental data (n = 15) in order to test our hypotheses within the identified parietal and occipital regions (i.e., to identify the presence of a transsaccadic feature-specific effect). For our first prediction (Fig. 2), these analyses were carried out for 15 participants who met our behavioural criteria (see *Methods: Behavioural data and exclusion criteria*; Fig. 3, orange) and the 12 participants who met our behavioural criteria for the saccade control task (see *Methods: Functional imaging data: saccade control task.*; Fig. 3, fuchsia). For our second prediction (Fig. 2), these analyses were carried out for the 15 participants who met our behavioural criteria.

Using the β-weights, we looked for specific directionality in the second prediction that we tested (Fig. 2), so we used one-tailed repeated measures t-tests (to identify feature-related specificity). For the frontal regions, where did not have a direction hypothesis, we tested feature sensitivity using two-tailed repeated measures t-tests. For the results of these analyses, we provided all relevant t-values, *p* values and effect sizes (Cohen’s d, determined using G*Power; Table 1)^69^.

## Supplementary Information

Supplementary Information
